# Oral crocetin administration suppressed refractive shift and axial elongation in a murine model of lens-induced myopia

**DOI:** 10.1038/s41598-018-36576-w

**Published:** 2019-01-22

**Authors:** Kiwako Mori, Toshihide Kurihara, Maki Miyauchi, Ayako Ishida, Xiaoyan Jiang, Shin-ichi Ikeda, Hidemasa Torii, Kazuo Tsubota

**Affiliations:** 1 Laboratory of Photobiology, Keio University School of Medicine, Shinjuku-ku, Tokyo 160-8582 Japan; 2 Department of Ophthalmology, Keio University School of Medicine, Shinjuku-ku, Tokyo 160-8582 Japan

## Abstract

Increased global incidence of myopia necessitates establishment of therapeutic approaches against its progression. To explore agents which may control myopia, we screened 207 types of natural compounds and chemical reagents based on an activity of a myopia suppressive factor, early growth response protein 1 (Egr-1) *in vitro*. Among the candidates, crocetin showed the highest and dose-dependent activation of Egr-1. For *in vivo* analysis, experimental myopia was induced in 3-week-old C57BL/6 J mice with −30 diopter (D) lenses for 3 weeks. Animals were fed with normal or mixed chow containing 0.003% (n = 19) and 0.03% (n = 7) of crocetin during myopia induction. Refraction and axial length were measured at 3-week-old and the 6-week-old with an infrared photorefractor and a SD-OCT system. Compared to controls (n = 14), crocetin administration showed a significant smaller change of refractive errors (−13.62 ± 8.14 vs +0.82 ± 5.81 D for 0.003%, p < 0.01, −2.00 ± 4.52 D for 0.03%, p < 0.01) and axial elongation (0.27 ± 0.03 vs 0.22 ± 0.04 mm for 0.003%, p < 0.01, 0.23 ± 0.05 mm for 0.03%, p < 0.05). These results suggest that a dietary factor crocetin may have a preventive effect against myopia progression.

## Introduction

The incidence of myopia is reported to be increasing in many countries over the past 50 years^1–3^. It has been reported that high myopia may cause severe ocular impairments as pathological myopia^4,5^. Investigation of preventive measures for progression of myopia is universally a crucial matter in the ophthalmologic field. Countermeasures against myopia control including topical atropine or pirenzepine administration, multifocal lens wearing, and orthokeratology have been reported^6^. Oral agents including dietary factors can also be candidates. To date, an adenosine antagonist 7-methylxanthine (7-Mx)^7,8^ has been reported to have an effect on myopia progression in children.

Although genetic factors are known to greatly influence the growth of the eye, fine correlation between the components of refraction for the eye to become emmetrope is affected by environmental factors such as education, diet, physical activity, and outdoor activity^9–12^. In the outdoor environment, we have reported that violet light, i.e. short wavelength in the visible light, has a suppressive effect against the progression in experimental myopia and school children and adult high myopia^13,14^. Among various myopia-related genes, *early growth response protein 1* (*Egr-1*) was shown to be upregulated by the violet light exposure both *in vivo* and *in vitro*^13^. Egr-1 is a transcriptional factor known as a myopia suppressive agent functioning in the feedback mechanism for axial ocular growth^13,15,16^. The gene knockout for *Egr-1* showed axial elongation in mice with additional minor effects on anterior chamber depth and corneal radius of curvature, and crystalline lens changes may underline the small difference in refractive error^17^. Therefore, *Egr-1* can be considered as a biological marker for myopia suppressive intervention.

There are two major types of experimental myopia; form-deprivation and lens-induced myopia^18,19^. By inducing myopia either by form deprivation or negative lenses, the effects of environmental cues and dietary habits on myopia development can be studied^18,19^. Form-deprivation leads to relatively weaker myopic phenotypes in mice than minus lenses, especially for axial elongation which sometimes dose not reach statistical significance^18,20^. Deprivation myopia is technically easier to handle than lens-induced myopia since lenses have to be kept clear and regularly cleaned while diffusers are frosted anyway. We have recently reported a robust and reproducible murine lens-induced myopia model with a newly designed skull-mounted eyeglass^20^. Combined with the measurement by a large focal depth spectral-domain optical coherent tomography, the lens-induced myopia mouse model showed a more significant phenotype than form-deprivation models. The model also reproduced a therapeutic effect of topical atropine treatment which was previously reported in other animal models and clinical trials^20^.

An interventional way to prevent myopia progression is in great demand, and dietary natural compounds would be safe and ideal especially for school children. In this study, we identified a dietary factor strongly inducing Egr-1 activity utilizing a luciferase-based reporter assay. We further confirmed a suppressive effect of the agent against a myopic change in the recently established murine model of lens-induced myopia.

## Results

### Crocetin induced a significant Egr-1 activation *in vitro*

**Figure 1:**
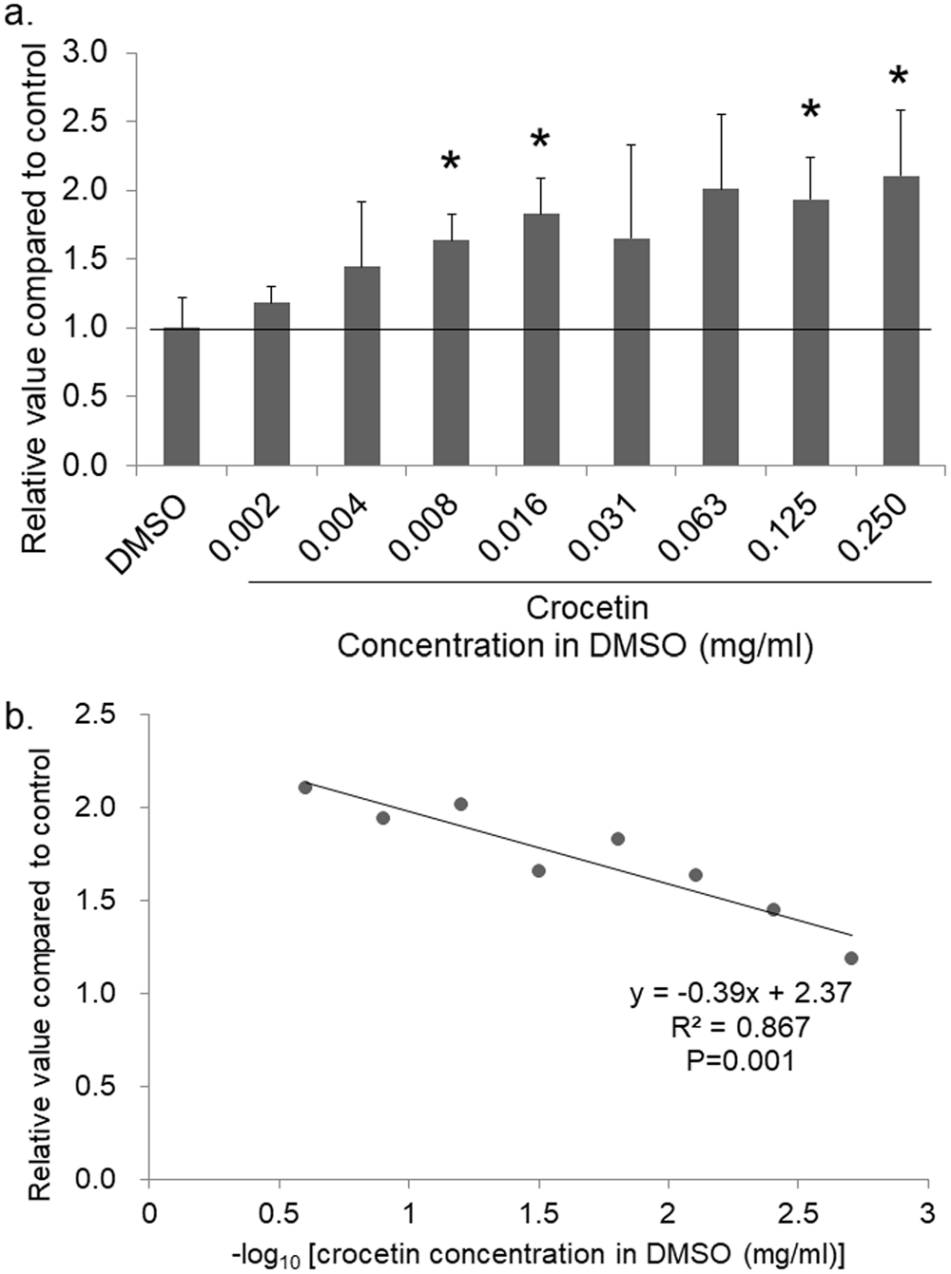
Comparison of relative values of Egr-1 activation at various concentrations of crocetin *in vitro*. (**a**) The assay of crocetin was repeated with various concentrations. Note that a statistically significant increase of Egr-1 activation was observed at least with 0.008 mg/ml. *p < 0.05. Bars represent mean +/− standard deviations. (**b**) Log-linear analysis revealed a significant linear correlation between the concentration of crocetin and the relative value of Egr-1 activation compared to control. X-axis is negative common logarithm of crocetin concentration in DMSO (mg/ml).

**Figure 2:**
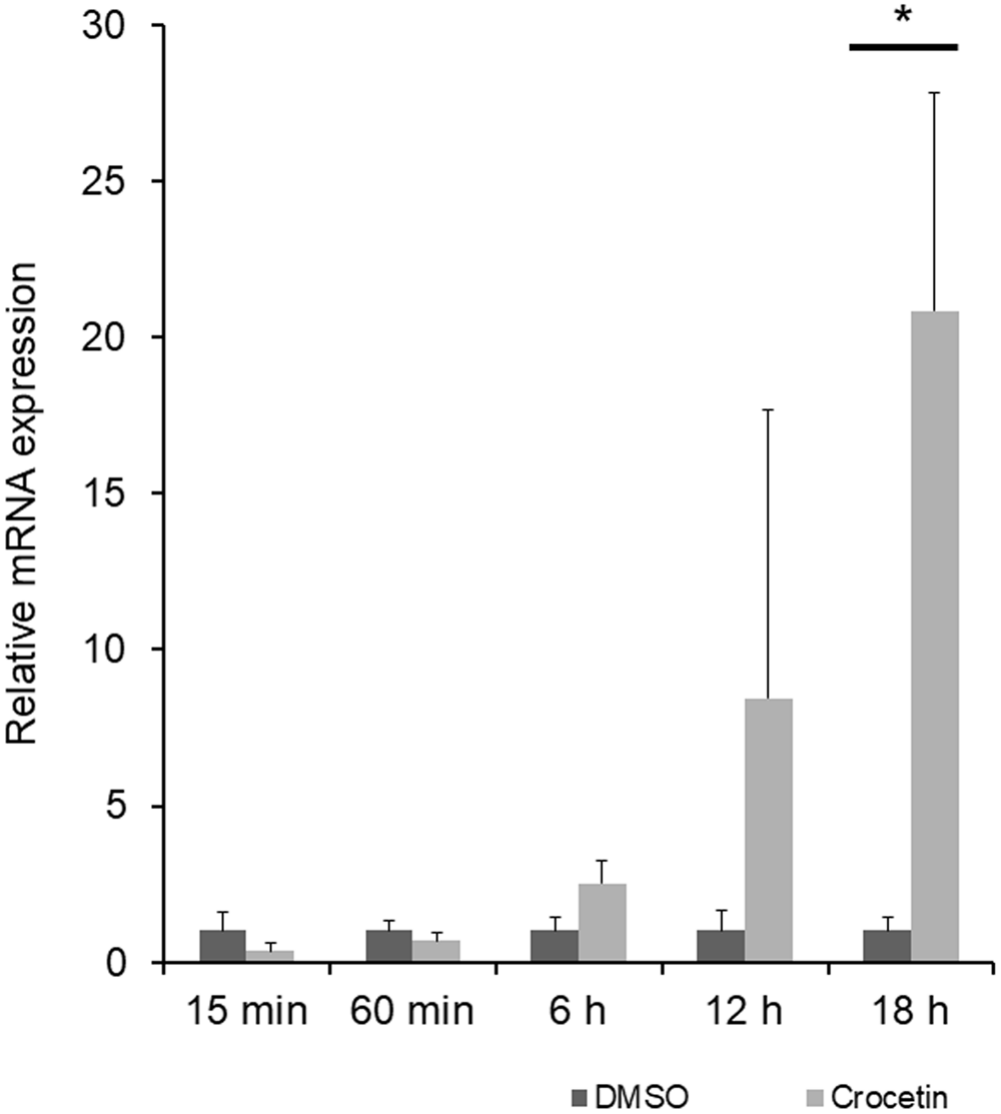
Relative expression of *Egr-1* mRNA was upregulated by crocetin administration. *Egr-1* expression was analyzed with real-time PCR adding crocetin to HEK293T cells. A significant increase of *Egr-1* mRNA expression was confirmed 18 hours after administration of crocetin. *p < 0.05.

Utilizing the Egr-1-luc cell line, 207 types of natural compounds and chemical reagents were examined. At the initial screening, 7 compounds showed more than 2.5-fold activation of Egr-1 compared to a negative control dimethyl sulfoxide (DMSO) without compounds. Among all candidates and a positive control phorbol 12-myristate 13-acetate (PMA)^21^, 75% or more purity of crocetin-containing gardenia fruit extract A (hereinafter called crocetin) showed the highest activation of Egr-1 (Supplementary Table 1). The secondary assay for crocetin was performed with various concentrations (0.002, 0.004, 0.008, 0.016, 0.031, 0.063, 0.125 and 0.25 mg/ml) (n = 3 for each). The Egr-1 activation with crocetin were 1.18 ± 0.12 folds for 0.002 mg/ml (p = 0.299), 1.45 ± 0.47 folds for 0.004 mg/ml (p = 0.230), 1.63 ± 0.19 folds for 0.008 mg/ml (p = 0.020), 1.83 ± 0.26 folds for 0.016 mg/ml (p = 0.013), 1.65 ± 0.68 folds for 0.031 mg/ml (p = 0.254), 2.01 ± 0.54 folds for 0.063 mg/ml (p = 0.058), 1.94 ± 0.30 folds for 0.125 mg/ml (p = 0.012), 2.1 ± 0.48 folds for 0.25 mg/ml (p = 0.036) (Fig. 1a). Log-linear analysis showed a statistically significant activation of Egr-1 with a dose-dependent manner (p < 0.001) (Fig. 1b). A significant upregulation of *Egr-1* mRNA was confirmed by real-time PCR 18 hours after administration of crocetin (Fig. 2).

### Oral administration of crocetin suppressed myopic shift in a murine model of lens-induced myopia

**Figure 3:**
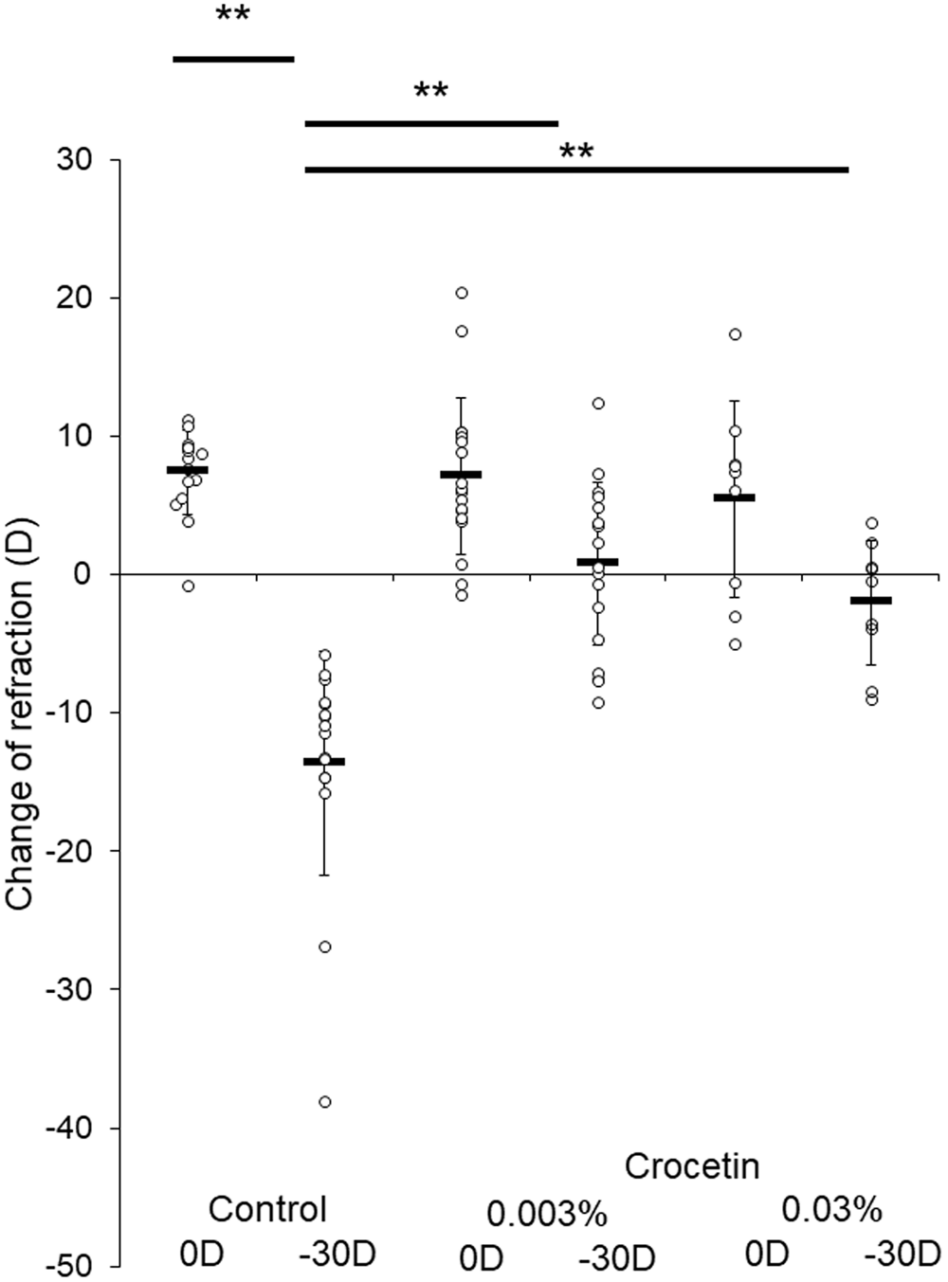
Crocetin suppressed myopic refractive shift in a murine model of lens-induced myopia. Eyes treated with −30 D lenses showed a significantly larger refractive change compared to ones with 0 D in the normal chow-fed animals (p < 0.001). Animals fed with 0.003% crocetin-containing chow showed a significantly smaller refractive change with −30 D lens compared to the same condition in the control (p < 0.001). Animals fed with 0.03% crocetin-containing chow showed a significantly smaller refractive change with −30 D lens compared to the same condition in the control (p < 0.001). The degrees of change of refraction were not significantly different between 0.03% crocetin with −30 D lenses and 0.003% crocetin with −30 D lenses. (p = 0.548). **p < 0.01, Bars represent mean +/− standard deviations.

**Figure 4:**
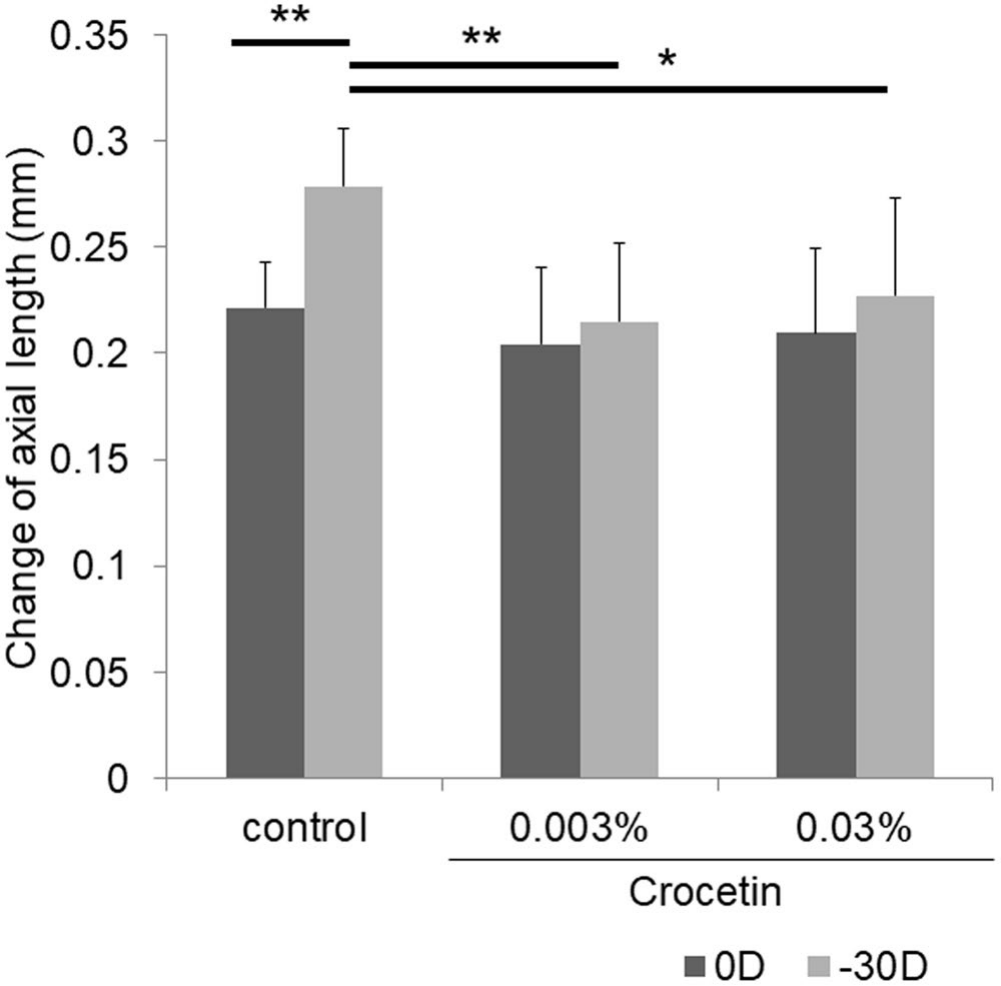
Crocetin suppressed axial elongation in a murine model of lens-induced myopia. The change of axial length of myopia-induced eyes with −30 D lenses showed a significantly more axial eye growth compared to control eyes with 0 D in the normal chow group (p < 0.001). The 0.003% and 0.03% crocetin chow group showed a significantly smaller axial length change in eyes with −30 D lenses compared to the normal chow group with −30 D lenses (p < 0.001 for 0.003%, p = 0.010 for 0.03%, respectively). The degrees of change of axial length were not significantly different between 0.03% crocetin with −30 D lenses and 0.003% crocetin with −30 D lenses (p = 0.510). *p < 0.05, **p < 0.01, Bars represent mean +/− standard deviations.

**Figure 5:**
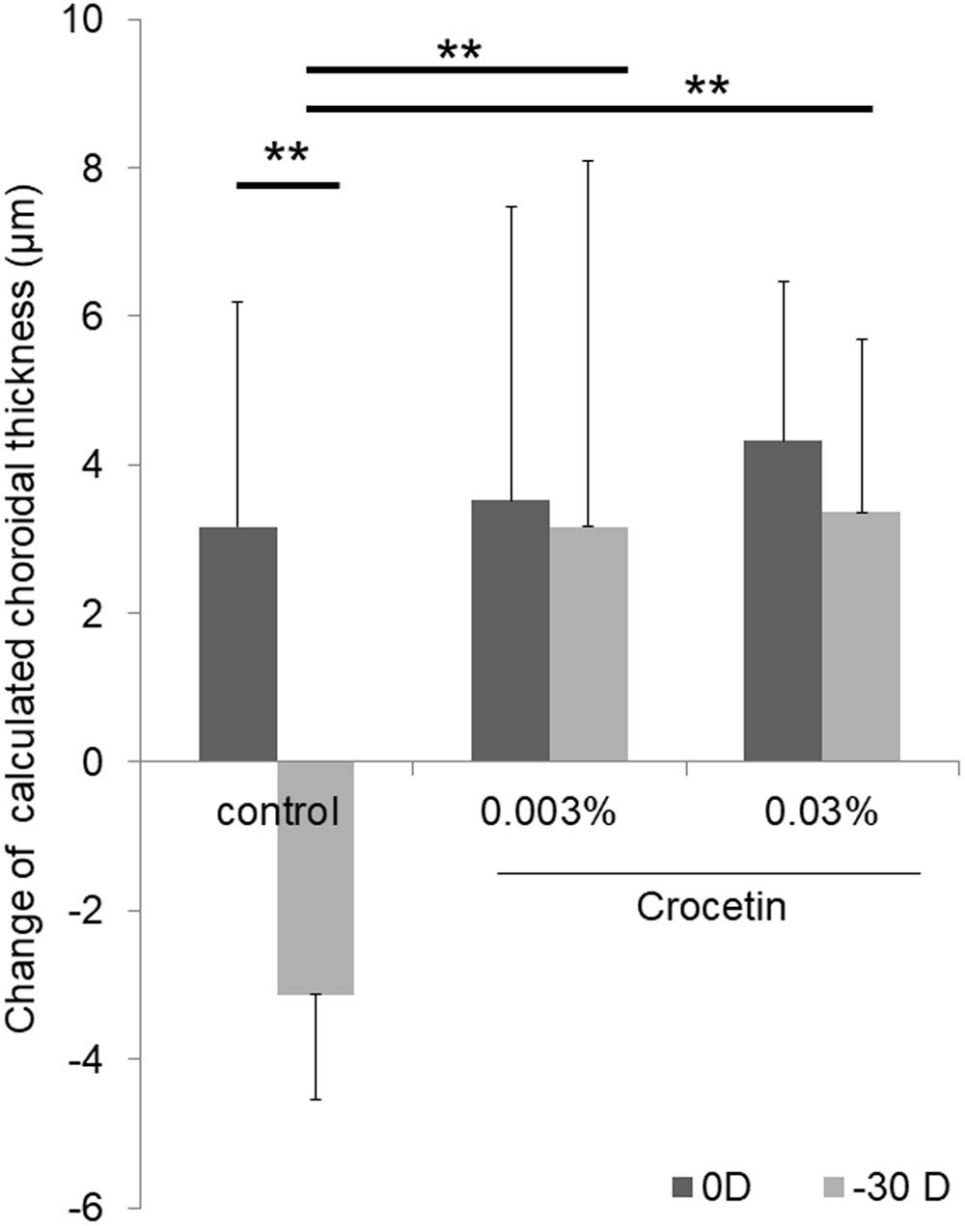
Decrease of choroidal thickness was diminished by crocetin administration. The comparison of change of choroidal thickness between myopia-induced eyes with −30 D lenses and control eyes with 0 D lenses showed significant difference in the normal chow group (p < 0.001). Mice fed with 0.003% and 0.03% crocetin chow with −30 D lenses showed positive change of choroidal thickness with a statistical significance (p < 0.001). **p < 0.01, Bars represent mean +/− standard deviations.

The candidate compound crocetin-containing chow was examined with the murine model of lens-induced myopia^20^. Animals were fed with either normal or two different concentrations (0.003% and 0.03%) of crocetin-containing chow. Eyes treated with -30 diopter (D) lenses showed a significantly larger refractive change (−13.62 ± 8.14 D) compared to ones with 0 D (+7.44 ± 3.04 D) in the normal chow-fed animals (p < 0.001, Fig. 3). Animals fed with crocetin-containing chow showed a significantly smaller refractive change with −30 D lens (+0.82 ± 5.81 D for 0.003%, −2.00 ± 4.52 D for 0.03%) compared to the normal chow group with −30 D (−13.62 ± 8.14 D) (p < 0.001 for both concentrations, Fig. 3). Eyes with −30 D lenses showed a significantly more axial eye growth (0.27 ± 0.03 mm) compared to ones with 0 D (0.22 ± 0.02 mm) in the normal chow group (p < 0.001, Fig. 4). The crocetin chow group also showed a significantly smaller axial length change in eyes with −30 D lenses (0.22 ± 0.04 mm for 0.003%, 0.23 ± 0.05 mm for 0.03%) compared to the normal chow group with −30 D lenses (0.27 ± 0.03 mm) (p < 0.01 for 0.003%, p < 0.05 for 0.03%, Fig. 4). Oral administration of crocetin showed suppression of refractive and axial length changes in the murine myopia model with two different concentrations, although there were no significant differences between the concentrations. Myopia-induced eyes with −30 D lenses showed a decrease (−3.13 ± 1.40 µm) of choroidal thickness and control eyes with 0 D lenses increased (3.17 ± 3.03 µm) choroidal thickness from the baseline, and these changes were significantly different (p < 0.001) in the normal chow group. This indicates that the choroidal thickness in lens-induced myopia becomes thin whereas choroidal thickness normally increases as a mouse grows without any intervention. In contrast, when mice were fed with 0.003% crocetin-containing chow, wearing −30 D lenses induced an increase (3.17 ± 4.93 µm) of choroidal thickness. When mice were fed with 0.03% crocetin-containing chow and put with −30 D lenses, an increase (3.36 ± 2.32 µm) of choroidal thickness was observed. Compared to the mouse fed with normal chow and equipped with –30 D lenses showing a decrease (−3.13 ± 1.40 µm) of choroidal thickness, mice fed with 0.003% and 0.03% crocetin chow showed positive change of choroidal thickness with statistical significance (p < 0.001) (Fig. 5). Corneal curvature radius showed no significant difference among each group (data not shown).

## Discussion

In this study, we focused on Egr-1^17,22^ as a myopia suppressive factor, and performed an *in vitro* screening assay with a dietary factor library using Egr-1 activity as an indicator. Out of 207 compounds, crocetin showed the highest and a statistically significant induction of Egr-1 activation (Supplementary Table 1). Although the intensity was different due to a variance in the product, a dose-dependent activation of Egr-1 was confirmed (Fig. 1). *Egr-1* mRNA upregulation was also confirmed significantly with real-time PCR (Fig. 2). Subsequently, we examined therapeutic effect of crocetin against myopia progression *in vivo*. Utilizing a murine model of lens-induced myopia which recently we developed, refraction and axial elongation changes were suppressed by oral crocetin administration (Figs 3 and 4).

Egr-1 is a transcriptional factor with three zinc-finger domains for sequence specific DNA-binding that belongs to the immediate early gene family. This molecule is involved with cell mitogenesis^23^, proliferation^24,25^, differentiation^26^, and apoptosis^27^ as context dependent manners^17^. It has also been reported to play a role in synaptic activation and long-term potentiation^28^. Egr-1 is reported to be a possible myopia suppressive factor which may affect axial eye growth, accompanying changes of other eye structures^13,15,22,29–31^.

Gardenia fruits are widely used in Asian countries as a natural colorant and as a Chinese traditional herbal medicine since they have homeostatic, hepatoprotective, analgesic, antiphlogistic, antipyretic, and hypolipidemic effects^32,33^. Geniposide, crocin, and crocetin are the major secondary metabolites in the fruit and have various pharmacological effects on different illness^33^. Crocetin is a unique apocarotenoid with C_20_ backbone and a carboxyl group at each end. The crocetin derivatives are responsible for the orange or red color of the fruit and are two active ingredients of this herbal medicine. In animal and human studies, it has been shown that crocetin exhibits a variety of pharmacological effects, such as antioxidant^34^, antihyperlipidemia^34^, antiatherosclerotic^35^, antiinflammatory^36^, antiproliferation^37^, neuroprotective effects^38^, insulin resistance improvement^39,40^, positive effects on sleep^41^, and attenuation of physical fatigue^42^, and prevent retinal degeneration^32,33,43,44^. The molecular weight of crocetin is relatively small compared to other carotenoids. Crocetin has a strong antioxidant effect inhibiting cellular oxidative damages mediated by reactive oxygen species derived from xanthine oxidase^45^.

Crocetin has been consumed as a natural product and its positive effects in health were suggested such as attenuation of physical fatigue^42^ and cardioprotection^35,46^. In terms of application for ocular diseases, crocetin and its relatives are now being investigated^44,47,48^. Among them, therapeutic effects against retinal conditions have been well documented including increase of blood flow^49^, suppression of pathological neovascularization^50^, and protection from photoreceptor and retinal ganglion cell degeneration^43,44,47,48^. Based on these mounting findings in animal models, conducted clinical trials have shown that dietary supplementation containing crocetin increased retinal sensitivity in age-related macular degeneration patients^44^. Orally administered crocetin is absorbed rapidly by the intestine and passed into the blood circulation, where it remains in the plasma as its intact free form or as glucuronide conjugates. Plasma crocetin concentration reaches its maximum within 0.5 h after oral administration in mice. The aqueous humor concentration was approximately 2 µM at 1.5 h after oral administration, which was a level sufficient to reduce *in vitro* cell death induced by H_2_O_2_ or tunicamycyn^43,48^. Orally administered crocetin at concentration of 100 mg/kg showed significant effect^43,48^. In our experiment, 0.003% mixed chow corresponding to 10 mg/kg as well as 0.03% mixed chow corresponding to 100 mg/kg showed the same degree of effect. The minimum concentration of crocetin showing a statistically significant increase of Egr-1 activation compared with control was 0.008 mg/ml (Fig. 1a) which corresponds to 24.4 µM according to the molecular weight. Yamauchi *et al*.^43^ reported that in their mouse experiment concentrations of plasma crocetin reached 109.6 µM 4 hours after oral administration of 100 mg/kg of the same purity of crocetin as in our experiment. In their rat experiment, the concentration of crocetin reached 62.5 µM in the plasma and 2.0 µM in the aqueous humor 1.5 hours after bolus oral administration of 50 mg/kg of crocetin. Since our *in vitro* study demonstrated that a concentration of 24.4 µM in the retina is minimally required to activate Egr-1, the level of crocetin in the retina might not reach the sufficient concertation for a sustained period during the oral administration. Therefore, in terms of Egr-1 activation, the *in vivo* experiments may be misleading because too low drug concentrations were used. Further study is necessary to know more exactly how much crocetin reaches into the retina after oral administration of crocetin.

To prove the suppressive mechanism of myopia progression, we examined *Egr-1* mRNA expression in the retina with real-time PCR, but the results were inconsistent. This may be because of that *Egr-1* expression can be changed rapidly with various external stimulations. The timing to take samples should have been more considered to obtain more precise results. Crocetin has been reported as a NMDA receptor antagonist, and stimulation of NMDA receptors often activates immediate early genes^51^. In our study, crocetin was demonstrated to increase *Egr-1* mRNA expression *in vitro* significantly in later but not early time points (Fig. 2). Since there is no direct correlation between retinal *Egr-1* expression and crocetin administration confirmed as discussed above, it is possible that a different mechanism is involved.

One possible targeted organ of crocetin for myopia suppression would be the choroid. Among various functions, the choroid is known to adjust the position of the retina by change of the thickness for emmetropization^52^. The choroidal thickness in myopic eyes is thinner than that of normal eyes which is associated with axial length in human^53^ and animal models^52^. In the current lens-induced mouse myopia model also showed a choroidal thinning together with myopic changes of the refraction and the axial length. Compared to the normal chow, feeding with crocetin prevented the choroidal thinning. Compared to the normal chow, feeding with crocetin prevented the choroidal thinning. Further studies are needed to examine whether this suppressive effect is dependent on *Egr-1* expression or not.

During recent decades, the prevalence rate of myopia has been dramatically increased worldwide^2^. Scientists and clinicians are now trying to explore and develop ways to control myopia especially in school children. To date, chemical compounds or light exposure increasing Egr-1 activity have been reported to suppress myopia progression in animal models^13,54^. Alternatively, our present study is the first to describe a natural extract crocetin as a myopia control agent through upregulation of *Egr-1*. To ensure the efficacy of crocetin, a prospective randomized control trial in the clinical setting is crucial and lately launched.

It has been reported that Egr-1 expression is associated with learning and neural plasticity^55–57^. We should know that crocetin has not only positive effects on the body such as neural function and ocular refraction but possible negative effects. In an adult experiment, no adverse effect was seen after oral administration of crocetin dosing 7.5–22.5 mg^58^. To the best of our knowledge, there have been no reports that tried administration of crocetin to children to see the effects on their neural development and its side effects. The minimal amount of crocetin to take effects, the dose of crocetin causing adverse effects, and the safety range of its dose in children should be more discussed and well examined.

## Materials and Methods

### Establishment of Egr-1 luciferase permanent expression cell line

HEK293T cells were maintained in Dulbecco’s Modified Eagle Medium (DMEM) high glucose (Nacalai, Japan #08456-65) supplemented with 10% Fetal Bovine Serum (FBS) (Invitrogen, USA #10270106), 100 units/ml Penicillin and 100 µg/ml Streptomycin (Nacalai, Japan #26253-84) at 37 °C in a 5% CO_2_ incubator. Cells were infected with Cignal Lenti Egr-1 reporter Luc (QIAGEN, Netherland #CLS-5021L) virus with SureENTRY Transduction reagent (QIAGEN, Netherland #336921) for less than 20 hours (25 MOI). After infection cells were passaged once, and then cultured under 10 µg/ml Puromycin (Sigma-Aldrich, USA #P8833) containing growth medium until no infected cell death completely. Five colonies were picked up and seeded in 2.0 × 10^4^ cells/well in HTS Transwell®-96 Receiver Plate, White, TC-Treated, Sterile (Corning, USA #3783). The highest reactive colony was used for the screening assay as the Egr-1-luc cell line in response to administration of a known Egr-1 activator phorbol 12-myristate 13-acetate (PMA: Promega, USA #V1171 or abcam, England #16561-29-8)^21^.

### *In vitro* screening assay with natural compounds and chemical reagents library

A total of 207 types of natural compounds and chemical reagents were prepared (Supplementary Table 1) and dissolved in dimethyl sulfoxide (DMSO) (Wako, Japan #043-07216) to the concentration of 100 mg/ml and settled overnight under dark places in room temperature after agitation. After centrifugation of 13,300 rpm for 3 minutes in room temperature, supernatant was added into the growth medium of the Egr-1-luc cell line. Each compound dissolved in DMSO was added to cell medium so that its concentration becomes 0.25 mg/ml (761.3 µM). Cells were incubated for 18 hours at 37 °C in a 5% CO_2_ incubator.

PMA of 100 nM was used for a positive control and so was DMSO-containing medium without any compounds for a negative control. Quantitation of the luciferase expression was performed utilizing ONE-Glo™ Luciferase Assay System (Promega, USA #E6110). One 10 ml Glo™ Luciferase Assay Buffer and 1 vial ONE-Glo™ Luciferase Assay Substrate of ONE-Glo™ Luciferase Assay System were mixed. In each well, we added 70 µL mixed liquid into the same volume medium. We read intensity of fluorescent at Synergy HTX (Biotek, USA) under the condition of shaking linear for 30 seconds, delay for 12 minutes, gain 180, integration time 0.5 seconds, and reading height 1.0 mm. The candidates selected by the method above were put under some concentrations and examined by a luciferase assay.

### Real time RT-PCR analysis

Crocetin (Crovit P, RIKEN VITAMIN Co., Ltd, Japan) dissolved in DMSO was added to HEK293T in cell medium so that its concentration becomes 0.25 mg/ml. Cells were incubated for 15 min, 60 min, 6 hours, 12 hours, and 18 hours at 37 °C in a 5% CO_2_ incubator. DMSO-containing medium without crocetin was used for a negative control. Total RNA was isolated from HEK293T using TRI reagent (MRC, USA #TR118) and Econospin column for RNA (GeneDesign, Japan #EP-21201). The columns were washed with Buffer RPE (QIAGEN, Netherland #1018013) and RWT (QIAGEN, Netherland #1067933). RNA was reverse-transcribed into cDNA using ReverTra Ace qPCR RT Master Mix) with gDNA remover (TOYOBO, Japan, #FSQ-301). SYBR green RT-PCR was performed using THUNDERBIRD SYBR qPCR Mix (TOYOBO, Japan, #QPS-201) and PCR was performed using StepOnePlus Real-Time PCR System (Applied Biosystems, USA). The relative amplification of cDNA fragments was calculated by the 2−ΔΔCt method. q-RT PCR primer sequences were as follows: human *Egr-1* forward: CTTCAACCCTCAGGCGGACA, human *Egr-1* reverse: GGAAAAGCGGCCAGTATAGGT, human GAPDH forward: TCCCTGAGCTGAACGGGAAG, human GAPDH reverse: GGAGGAGTGGGTGTCGCTGT.

### Animals

All procedures were performed in accordance with the National Institutes of Health (NIH) guidelines for work with laboratory animals and the ARVO Animal Statement for the Use of Animals in Ophthalmic and Vision Research, and were approved by the Institutional Animal Care and Use Committee at Keio University. C57BL6/J mice (CLEA Japan, Japan) were raised in standard transparent mouse cages (29 × 18 × 13 cm) in an air-conditioned room maintained at 23 ± 3 °C under a 12-h dark/light cycle, with free access to a standard diet (MF, Oriental Yeast Co., Ltd, Japan) and tap water. Four or five mice were kept in one cage with approximately 50 lux background fluorescent lamp light with 12 hour on/off. The intensity and cycle of the illumination was chosen based on the condition in which we established and reported the murine model of experimental myopia^20^.

Our study was also in compliance with ARRIVE guidelines. Assignment of the animals to each group was as follows; each animal was randomly assigned to one of the groups, 0.003% crocetin group, 0.03% crocetin group, and control group, in the completely blind manner. The mice were numbered to hide the treatment status. The researchers who bled the mice and ones who measured the refractive error were different. Therefore, the researchers were completely masked to the treatment status of animals during measurements of refractive error. Due to the variability, mice with the same sex (male) and the same body weight (10 ± 1 gram) were prepared.

### *In vivo* analysis with the dietary factor in a murine model of lens-induced myopia

For the *in vivo* analysis, we performed the experiment of a lens-induced myopia in mice as previously reported^20^. Briefly, we designed a frame of eyeglasses for mice accommodating to the shape of the mouse head and outputted it using a three-dimensional printer. For myopia induction, a negative 30 diopter (D) lens made of PMMA was designed and the side of the frame was fit to equip the right eye with the lens. A 0 D lens was fixed to the left eye as an internal control. Three-week-old wild-type C57BL/6 J mice were equipped with the eyeglasses by mounting the frame on their skull using a self-cure dental adhesive system under general anesthesia with the combination of midazolam (Sandoz K.K., Japan), medetomidine (Domitor®, Orion Corporation, Finland) and butorphanol tartrate (Meiji Seika Pharma Co., Ltd., Japan) (MMB). A 0.01 ml/g of MMB was administered intraperitoneally. Animals were fed with normal (MF, Oriental Yeast Co., Ltd, Japan) or mixed chow containing the candidate compound 0.003% and 0.03% crocetin (Crovit P, RIKEN VITAMIN Co., Ltd, Japan) during the period of the myopia induction. Crocetin was added by the chow manufacturing company (Oriental Yeast Co., LTD., Japan). A total of 3.6 g of Crovit P with the purity of more than 75% of crocetin was added to 11996.4 g of MF and dissolved to water. It was desiccated after molding. The total amount of the product was 12 kg and the concentration of crocetin was 0.03%. A total of 0.369 g of the same product was added to 110 g of MF. Then, 100.36 g of this compound was added to 11899.64 g of MF and dissolved in water. It was desiccated after molding. The total amount of the product was 12 kg and its concentration of crocetin was 0.003%. The concentration was measured and confirmed after these processes. The number of mice was four in each group. With regard to ideal concentration of crocetin administered orally was determined by calculating from average body weight and average amount of intake of 3-week-old mice referring to studies which suggest its concentration range from 10 mg/kg to 100 mg/kg^43,59^.

### Ocular components measurement

Refraction and axial length were measured at the initial (3-week-old) and the end (6-week-old) stage of the myopic induction using an infrared photorefractor (Steinbeis Transfer Center, Germany) and a SD-OCT system (Envisu R4310, Leica, Germany). All measurements were performed under mydriasis by 0.5% tropicamide and 0.5% phenylephrine eye drops (Santen, Japan), and general anesthesia by MMB. The refractive error values were averaged with 100 times of the measurement in a continuous data trace. The axial length was determined from the anterior corneal surface to the retinal pigment epithelium along the corneal vertex reflection. Since the choroidal thickness was considered to vary by the location, the choroidal area distant from the disc was calculated to compare the mean choroidal thickness. The area of the circumference at 0.5 mm from the disc circled at the border of the retinal pigment epithelium and the posterior surface of the choroid^60^ was quantified with ImageJ (National Institutes of Health, Bethesda, USA) (Supplementary Fig. 1). Choroidal thickness is led by the formula; the area is divided by the circumference. The corneal curvature radius was measured by an infrared keratometer (Steinbeis Transfer Center, Germany).

### Statistical analyses

All results are expressed as mean ± standard deviation (SD). Independent t-test was used to assess the statistical significance of the differences (Microsoft Excel 2013, USA), and results with P-values < 0.05 were considered significant.

## Electronic supplementary material

Supplementary Information

## Data Availability

The authors confirm that all data underlying the findings are fully available without restriction.
