# Supplementary material for: Oral crocetin administration suppressed refractive shift and axial elongation in a murine model of lens-induced myopia
Source: Sci Rep. 2019 Jan 22;9:295. doi: 10.1038/s41598-018-36576-w (PMC6343000; doi:10.1038/s41598-018-36576-w)
Supplement: Supplementary file 1 — Supplementary Information [file 41598_2018_36576_MOESM1_ESM.docx]

**Supplementary Information for**

**Oral crocetin administration suppressed refractive shift and axial elongation in a murine model of lens-induced myopia**

Kiwako Mori^1,2^, Toshihide Kurihara^1,2*^, Maki Miyauchi^1,2^, Ayako Ishida^1,2^, Xiaoyan Jiang^1,2^, Shin-ichi Ikeda^1,2^, Hidemasa Torii^1,2^, Kazuo Tsubota^1*^

^1^Laboratory of Photobiology, ^2^Department of Ophthalmology, Keio University School of Medicine

Table of Contents:

Supplementary Table 1

Supplementary Figures 1

**Supplementary Table 1. The list of natural compounds and chemical reagents used for the *in vitro* screening assay and the relative Egr-1 activity**

| Products | Relative intensity |
| --- | --- |
| DMSO | 1.00 |
| PMA | 2.54 |
| Gardenia fruit extract A (crocetin ≥75%) | 5.72 |
| Ginkgo biloba extract B (total flavonoid glycosides ≥24%, total terpene lactones ≥6%) | 3.80 |
| Ginkgo biloba extract H (total flavonoid glycosides ≥24%, total terpene lactones ≥6%, ginkgolide A ≥1.2%, ginkgolide B ≥0.8%, ginkgolide C ≥1.0%, bilobalide ≥2.5%) | 3.44 |
| Ginkgo biloba extract L (total flavonoid glycosides ≥24%, total terpene lactones ≥6%, ginkgolide B ≥0.8%, ginkgolic acid ≥5ppm) | 3.36 |
| Hovenia dulcis extract | 3.26 |
| Pearl barley seed extract | 3.11 |
| Ginkgo biloba BG extract D | 3.08 |
| Ginkgo biloba extract C (total flavonoid glycosides ≥24%, total terpene lactones ≥6%) | 2.98 |
| Walnut seedcoat extract A (polyphenol ≥10%, Tellimagrandin ≥0.1%) | 2.93 |
| Parsley extract | 2.87 |
| Ginkgo biloba extract A (total flavonoid glycosides ≥24%, total terpene lactones ≥6%) | 2.83 |
| Sudachi peel extract (total sudachitin ≥1%, total polyphenol ≥2%) | 2.76 |
| Strawberry seed extract (polyphenol ≥2%, tiliroside ≥0.5%) | 2.74 |
| Mangosteen peel extract B (Maclurin glycoside ≥0.03%) | 2.74 |
| Broccoli sprout extract A (Sulforaphane ≥1.2%) | 2.65 |
| Lactoferrin | 2.65 |
| Guarana seed extract (caffeine 22-26%) | 2.61 |
| Ginkgo biloba extract G (total flavonoid glycosides ≥24%, total terpene lactones ≥6%) | 2.56 |
| Oil droplets made from palm oil and oat oil | 2.53 |
| Peptide formulation derived from dairy protein (proteins ≥80%) | 2.52 |
| Bangle (Zingiber purpureum) extract | 2.48 |
| Citrus extract B (flavanones ≥20%, caffeine 1-4%) | 2.48 |
| Calcium Ascorbate | 2.48 |
| Gardenia fruit extract F (crocin ≥20%) | 2.42 |
| Mulberry leaf extract A | 2.40 |
| Ginkgo biloba BG extract O | 2.38 |
| Perilla seed extract A (polyphenol ≥3.0%) | 2.33 |
| Quercus salicina leaf extract B | 2.33 |
| Ginkgo biloba BG extract M | 2.28 |
| Coenzyme Q10 | 2.26 |
| Kiwi seed extract B (polyphenol ≥2.0%, quercitrin ≥5mg/100g, water soluble) | 2.22 |
| American ginseng extract | 2.18 |
| Cyanocobalamin | 2.15 |
| Spatholobus suberectus extract | 2.10 |
| Cistanche salsa extract | 2.09 |
| Safflower extract | 2.01 |
| Assai fruit extract | 2.00 |
| Marigold flower extract A (lutein ≥10%, zeaxanthin ≥0.6%) | 1.97 |
| Lonicera caerulea fruit extract B (cyanidin 3-glucoside ≥8%) | 1.93 |
| Jujube fruit extract | 1.81 |
| Gardenia fruit BG extract P | 1.76 |
| Ajuga extract | 1.72 |
| Kaempferia parviflora extract （5,7-dimethoxyflavone ≥4%, total polymethoxyflavonoids 15%) | 1.71 |
| Thiamine Mononitrate | 1.68 |
| German chamomile extract | 1.67 |
| Tangerine peel extract B | 1.64 |
| Ginkgo biloba extract I (total flavonoid glycosides ≥24%, total terpene lactones ≥6%, gingkolic acid ≤1ppm) | 1.62 |
| Guarana extract | 1.62 |
| Red spinach extract （nitrate nitrogen ≥9%) | 1.61 |
| Houttuynia extract | 1.61 |
| Calcium D-Pantothenate | 1.59 |
| Bitter orange extract | 1.58 |
| Ginkgo biloba ET extract N | 1.58 |
| Perilla seed extract B (polyphenol ≥3.0%, water soluble) | 1.54 |
| Grape pomace extract A (Oleanolic Acid ≥3.0%) | 1.54 |
| Soybean extract B (total isoflavones ≥40%) | 1.54 |
| Thioctic acid (alpha lipoic acid) | 1.50 |
| Viper extract | 1.50 |
| Gardenia fruit extract C | 1.50 |
| Plant mixed extract | 1.48 |
| Eleutherococcus senticosus root extract | 1.46 |
| Apocynum venetum leaf extract | 1.43 |
| Fucoidan | 1.42 |
| Vitamin A Palmitate | 1.42 |
| Ginkgo biloba extract K (total flavonoid glycosides 15-35%, total terpene lactones 4-12%, Ginkgolic acid ≤10ppm) | 1.42 |
| Bird's nest extract | 1.41 |
| Longan extract | 1.41 |
| Walnut seedcoat extract C (polyphenol ≥10%, Tellimagrandin ≥0.1%, water soluble) | 1.39 |
| Quercus salicina leaf extract A (total tannin ≥18%) | 1.39 |
| Reindeer horn extract | 1.39 |
| Cats whiskers extract | 1.38 |
| Tangerine peel ET extract A | 1.36 |
| Gardenia fruit extract G | 1.36 |
| β-Carotene 1% A | 1.35 |
| Ginkgo biloba extract F (total flavonoid glycosides ≥24%, total terpene lactones ≥6%, ginkgolide A ≥1.0%, ginkgolide B ≥0.8%, ginkgolide C ≥0.6%, bilobalide ≥2.0%) | 1.34 |
| Tangerine peel BG extract E | 1.32 |
| Cistanche tubulosa extract | 1.30 |
| Tian cha leaf extract | 1.26 |
| Salacia extract | 1.25 |
| D-Biotin | 1.24 |
| Eucommia leaf extract | 1.22 |
| Pfaffia extract (20-Hydroxyecdysone ≥1%) | 1.22 |
| Platycodon grandiflorum root extract | 1.20 |
| Banaba leaf extract | 1.18 |
| Kabosu seed extract (total limonoids ≥2%) | 1.18 |
| Mulberry leaf extract B | 1.15 |
| Gardenia fruit extract K | 1.14 |
| Camu camu fruit extract (vitamin C ≥12%) | 1.13 |
| Cloudy bud extract | 1.13 |
| Perilla leaf extract (Luteolin ≥0.6%) | 1.10 |
| Saffron stigma extractt | 1.10 |
| Gardenia fruit BG extract I | 1.10 |
| Yacon extract | 1.08 |
| Linseed (claxseed) extract (lignan ≥40%） | 1.07 |
| Soybean extract A (isoflavone ≥37%) | 1.04 |
| Vitamin D3 | 1.04 |
| Rhodiola rosea extract (Salidroside ≥3%) | 1.04 |
| Lonicera caerulea fruit extract A | 1.03 |
| Dandelion root extract | 1.03 |
| Royal jelly extract | 1.03 |
| Ascophyllum nodosum extract (polyphenol ≥75%) | 1.02 |
| Grape extract (polyphenol ≥25%, resveratrol ≥5%） | 1.00 |
| Lotus root extract | 0.99 |
| Ginkgo biloba extract J (gingkolic acid ≤5ppm) | 0.98 |
| Paprica extract A | 0.97 |
| Grape pomace extract B (Oleanolic Acid ≥2.0%) | 0.93 |
| Silybum marianum fruit extract | 0.93 |
| Citrus peel extract | 0.93 |
| Ginkgo biloba extract Q (total flavonoid glycosides ≥24%, total terpene lactones ≥6%) | 0.92 |
| Silybum marianum extract (silymarin ≥80%, silybin ≥30%) | 0.90 |
| Perilla seed extract C (polyphenol ≥2.0%) | 0.89 |
| Lotus leaf extract | 0.89 |
| Glossy privet fruit extract | 0.89 |
| Glucosyl hesperidin (total hesperidin ≥70%) | 0.89 |
| Garcinia peel extract B (hydroxycitric acid ≥60%) | 0.88 |
| Panax notoginseng extract | 0.88 |
| Hesperetin | 0.87 |
| Thiamine Hydrochloride | 0.85 |
| Methyl hesperidin | 0.85 |
| Fruit and vegetable extract | 0.83 |
| Melinjo seed extract (resveratrol ≥20%) | 0.82 |
| Broccoli extract | 0.82 |
| Blueberry leaf extract (total polyphenol ≥30%) | 0.79 |
| Siberian larch extract (Dihydroquercetin ≥88%) | 0.79 |
| Evening primrose seed extract (polyphenol ≥60%) | 0.79 |
| Panax ginseng root extract | 0.78 |
| Orange peel extract | 0.76 |
| Mangosteen peel extract A (alpha-mangostin ≥20%) | 0.76 |
| Chestnut peel extract | 0.76 |
| Crataegus oxycanthal extract | 0.76 |
| Tangerine peel powder | 0.76 |
| Grape bud extract (total resceratrol ≥20%, trans-resceratrol ≥5%, epsilon-viniferin ≥5%) | 0.75 |
| β-Carotene 1% B | 0.75 |
| Grape seed extract　B | 0.75 |
| Seahorse (Hippocampus) extract | 0.74 |
| Plantago asiatica extract | 0.73 |
| Lingonberry extract A (trans-resveratrol ≥10％, anthocyanin ≥10%) | 0.72 |
| Olive fruit extract (Maslinic acid ≥10%) | 0.72 |
| Echinacea extract | 0.67 |
| Ginkgo biloba extract E (total flavonoid glycosides ≥25%, total terpene lactones ≥6%, Ginkgolic acid ≤5ppm) | 0.66 |
| Bilberry fruit extract A (total anthocyanosides ≥85%) | 0.65 |
| β-Carotene 5% | 0.64 |
| Black ant (Polyrhachis vicina) extract | 0.64 |
| Curcuma extract C (total curcuminoids 18-22%, curcumin ≥13%) | 0.63 |
| Pyridoxine Hydrochloride | 0.59 |
| Maqui berry fruit extract (anthocyanins ≥35%, delphinidins ≥20%） | 0.57 |
| Gardenia fruit extract O | 0.56 |
| Garcinia peel extract A (hydroxycitric acid 42±2%, water soluble) | 0.54 |
| Isatis tinctoria extract | 0.54 |
| Polygonatum falcatum root extract | 0.53 |
| Ascorbyl Palmitate | 0.52 |
| Vitamin D3 | 0.52 |
| Paprica extract C (tottal xanthophyll ≥9mg/g, capsanthin ≥5mg/g, beta-cryptoxanthin ≥0.5mg/g) | 0.50 |
| French marine pine bark extract (procyanidin 65-75%) | 0.50 |
| Walnut seedcoat extract B (polyphenol ≥30%, Tellimagrandin ≥0.3%) | 0.48 |
| Gardenia fruit extract B | 0.48 |
| Tangerine peel ET extract D | 0.48 |
| Grape seed extract A (proanthocyanidin ≥95%) | 0.47 |
| Cinnamon peel extract | 0.47 |
| Ganoderma lucidum extract | 0.46 |
| Black soybean extract (polyphenol ≥58%) | 0.44 |
| Black currant fruit extract (anthocyanin ≥35%) | 0.44 |
| Perilla leaf extract | 0.44 |
| Green tea leaf extract (epigallocatechin gallate ≥55%) | 0.43 |
| Guarana seed extract | 0.42 |
| Cornus officinalis extract | 0.39 |
| Citrus extract A | 0.38 |
| Curcuma extract D | 0.38 |
| Kiwi seed extract A (polyphenol ≥2.0%, quercitrin ≥5mg/100g) | 0.35 |
| Sodium Ascorbate | 0.33 |
| β-Carotene 30% A | 0.33 |
| β-Carotene 30% B | 0.31 |
| Aronia fruit extract (triterpenic acids ≥6%) | 0.30 |
| Red peppers extract | 0.30 |
| Gardenia fruit extract M | 0.30 |
| Broccoli sprout extract B (Sulforaphane ≥2.0%) | 0.28 |
| Ginkgo biloba extract P | 0.28 |
| Riboflavin | 0.26 |
| Psidium guajava leaf extract | 0.24 |
| Bilberry fruit extract B (total anthocyanosides ≥85%) | 0.24 |
| Niacinamide | 0.22 |
| Curcuma extract B (tetra hydro curcuminoid ≥95%) | 0.20 |
| Riboflavin 5'-Phosphate Sodium | 0.18 |
| Corn silk stylar extract | 0.17 |
| Curcuma extract A (total curcuminoid content ≥95%) | 0.15 |
| Marigold flower extract B (lutein ≥20%, zeaxanthin 1-2%) | 0.15 |
| Lychee fruit extract | 0.14 |
| Gardenia fruit extract D | 0.12 |
| epsilon-viniferin | 0.09 |
| Gardenia fruit extract E | 0.08 |
| Gnetin C | 0.06 |
| trans-resveratrol | 0.02 |
| Lingonberry extract B (arbutin ≥0.5%) | 0.00 |
| Amla fruit extract (gallotannin ≥15%） | 0.00 |
| Mallotus japonicus peel extract (Bergenin ≥12%) | 0.00 |
| Mangosteen extract (alpha-mangostin ≥10%, gamma-mangostin ≥1%) | 0.00 |
| Paprica extract B (tottal xanthophyll ≥27mg/g, capsanthin ≥15mg/g, beta-cryptoxanthin ≥0.5mg/g) | 0.00 |
| Ginger extract | 0.00 |
| Gardenia fruit extract H | 0.00 |
| Tangerine peel ET extract F | 0.00 |
| Gardenia fruit ET extract J | 0.00 |
| Gardenia fruit extract L | 0.00 |
| Folic acid | N/A |
| Oat beta-glucan A (27-29% beta-glucan) | N/A |
| Oat beta-glucan B (13-15% beta-glucan) | N/A |
| Oat beta-glucan C (21-23% beta-glucan) | N/A |
| Gardenia fruit extract N | N/A |

**Supplementary Figure1:**

~~
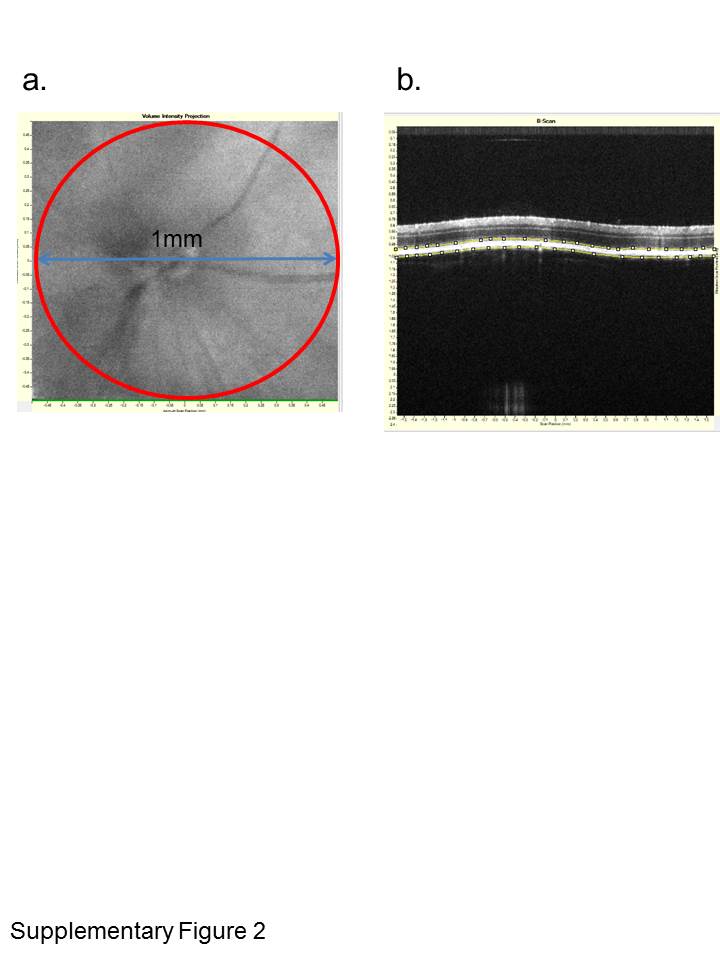
~~

**Supplementary Figure 1: Quantification method of choroidal thickness utilizing the SD-OCT system**

a. A fundus photography captured in the OCT system. The red circle indicates the circumference at 0.5 mm from the disc.

b. A cross sectional image of the circumference captured in the OCT system. The area of the circumference at 0.5 mm from the disc circled at the border of the retinal pigment epithelium and the posterior surface of the choroid was quantified with ImageJ.
